# Lipoma of the Small Intestine: A Cause for Intussusception in Adults

**DOI:** 10.1155/2015/856030

**Published:** 2015-07-29

**Authors:** Ketan Vagholkar, Rahulkumar Chavan, Abhishek Mahadik, Inder Maurya

**Affiliations:** Department of Surgery, D.Y. Patil University School of Medicine, Navi Mumbai, Maharashtra 400706, India

## Abstract

Intussusception as a cause of intestinal obstruction in adults is rare. There is invariably an underlying pathology which leads to intussusception in adults. A case of intussusception in an adult due to a small intestinal lipoma is presented in view of this association. Ultrasound and CECT may help in a preoperative diagnosis. However early surgical intervention is the mainstay of treatment in order to confirm the diagnosis of the underlying pathology, thereby avoiding misdiagnosis of an underlying cancer.

## 1. Introduction

Intussusception is defined as telescoping of one segment of bowel into another one. It is an uncommon cause of intestinal obstruction in adults with a reported incidence of 1 in 1300 abdominal cases presenting as obstruction [1]. It is predominantly seen in the paediatric population whereas in adults it is usually associated with various pathological lesions which serve as an apex for intussusception. Submucosal lipoma of the small intestine which is otherwise a silent pathology sometimes manifests clinically as acute intussusception. A case of a 22-year-old male patient who presented with acute intussusception due to small bowel lipoma is reported.

## 2. Case Report

**Figure 1:**
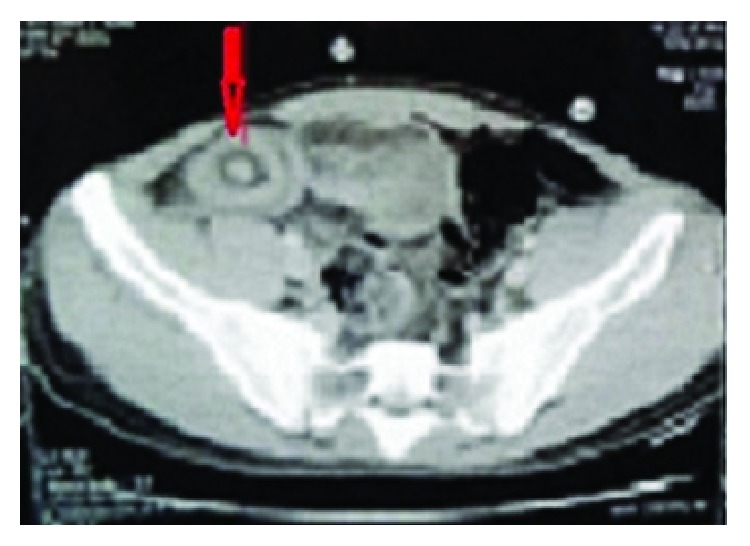
CECT showing typical target sign.

**Figure 2:**
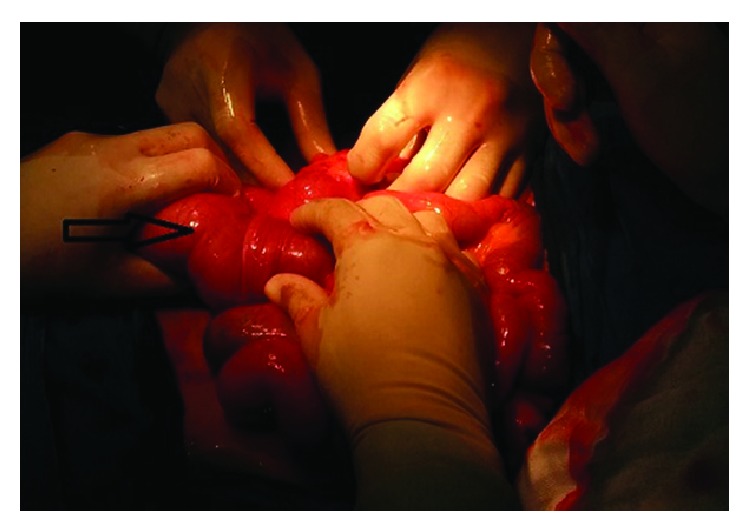
Attempt at reduction of the intussusception in the retrograde direction marked by the black arrow.

**Figure 3:**
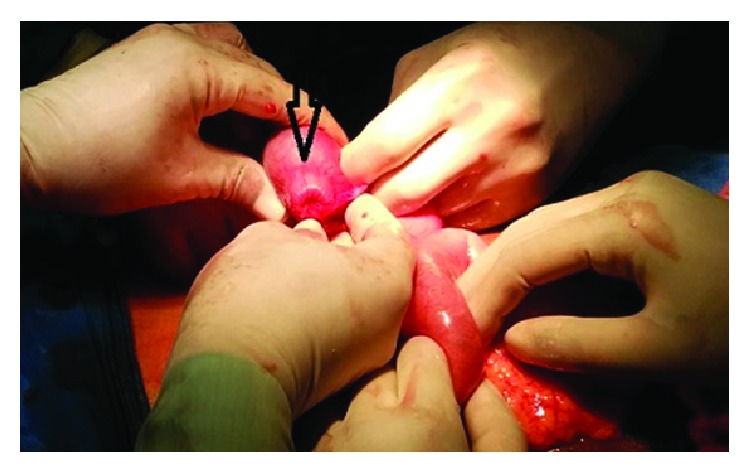
Invagination and depression of the serosal surface overlying the pathological lead point.

**Figure 4:**
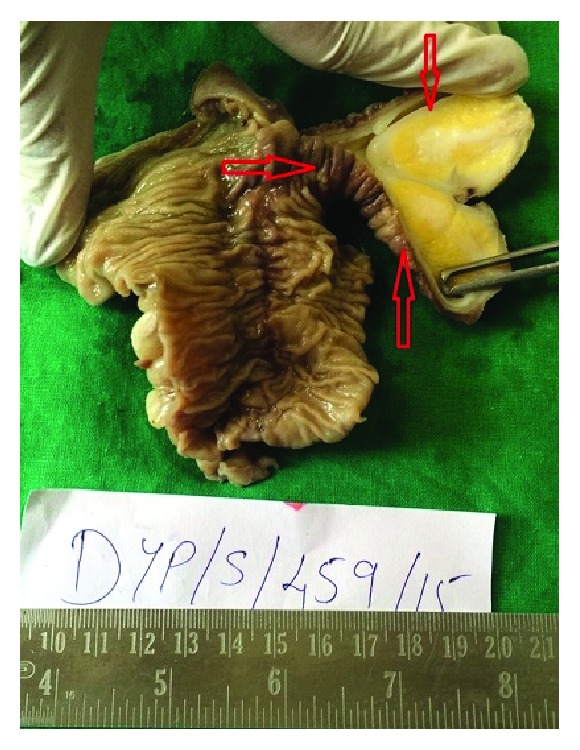
Specimen cut open revealed a pedunculated lipoma of the small bowel.

**Figure 5:**
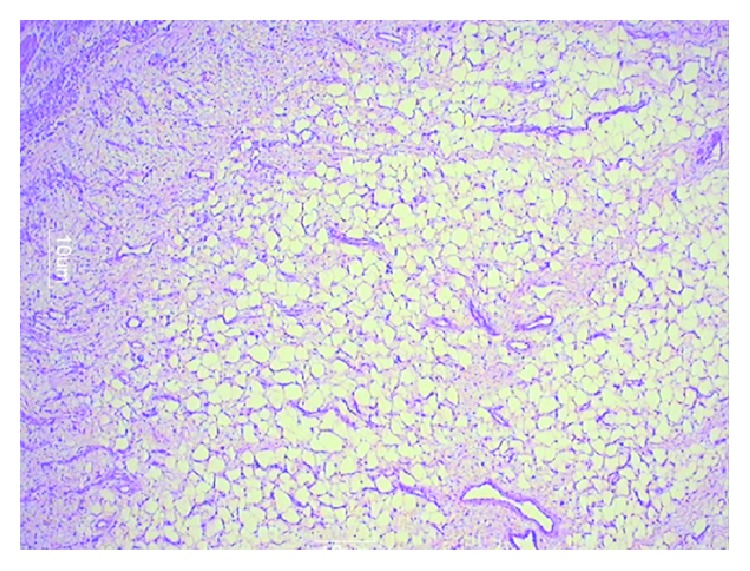
Histopathological features typical of a benign submucosal lipoma (H&E staining with magnification of 40x).

A 22-year-old male patient presented with clinical features of severe colicky pain in the right side of the abdomen accompanied with vomiting for two days. The patient gave a history of intermittent colicky pain in right lower abdomen over the last one month. All the vital parameters were within normal limits. Physical examination revealed a tender lump in the right lower quadrant of the abdomen. The lump disappeared after some time and then again reappeared with the onset of symptoms. Per rectal digital examination revealed blood stained mucus. After admission primary resuscitation was done to correct the fluid and electrolyte imbalance. The patient passed red currant jelly-like stools few hours after admission. X-ray of the abdomen was unremarkable at presentation. However ultrasonography revealed pseudokidney sign. Contrast enhanced CT of abdomen revealed the typical target sign in the right lower abdomen suggestive of intussusception with an intraluminal homogenous hypodense lesion at the apex of intussusception most likely suggestive of lipoma (Figure 1). The patient underwent exploratory laparotomy. Laparotomy revealed an ileoileocolic intussusception with the apex point almost reaching the hepatic flexure of colon. With utmost gentleness intussusception was reduced by the traditional method of milking from distal to proximal direction after having released the adhesions at the neck of the lesion (Figure 2). After reduction an intraluminal mobile lump was palpable approximately 2 feet proximal to ileocecal junction causing invagination and depression over serosa due to pulling from inside which was the pathological lead point for intussusception (Figure 3). Resection anastomosis of the involved segment of the bowel with a 5 cm margin on either side was done. Cut section of resected specimen showed a pedunculated polyp with intact overlying mucosa, typical of a submucosal lesion (Figure 4). Histopathological examination of the specimen confirmed the diagnosis of a benign submucosal lipoma (Figure 5). Postoperative course was uneventful. The patient has followed up for three months and is completely symptom free.

## 3. Discussion

Intussusception is a leading cause of intestinal obstruction in the paediatric population whereas in adults it is a rare entity which accounts for only 1–5% of all cases presenting as bowel obstruction. Unlike children, in majority of adult patients presenting with intussusception, the underlying pathological condition can be found [2]. Pathological conditions like carcinoma, lipoma, lymphoma, diverticulum, and adenomatous polyp act as a lead point and produce invagination within lumen, thus predisposing to intussusception. A high index of suspicion is justified while dealing with intussusception in adults. The incidence of malignancy in small bowel lesions presenting as intussusception is approximately 30 percent [1]. Based on the part of the bowel involved, intussusception has been traditionally classified into four types as follows: (1) ileoileal (confined to small bowel), (2) colocolic (involving the large bowel), (3) ileocolic (terminal ileum prolapsing into the ascending colon), and (4) ileocecal (the ileocecal valve is the leading point) [3, 4].

Lipoma is a ubiquitous benign tumour which is found anywhere along the GI most commonly in the ileum [5]. Smaller lesions do not cause many symptoms. However larger lesions invariably lead to intussusception. The terminal ileum is the commonest site for lipomas to occur. The submucosal type is most common followed by intermuscular and serosal types.

Preoperative diagnosis of intussusception in adults is a very challenging problem due to variability in the clinical presentation. As in the case presented careful repeated clinical examinations will help in establishing the intermittency of the abdominal lump coinciding with the patient's symptoms of bowel obstruction. Reijnen et al. [6] reported that preoperative diagnosis could be made only in 50% of patients with the help of imaging studies. Ultrasonography (US) and computed tomography (CT) of abdomen are very helpful imaging modalities for diagnosis of intussusception and nature of intraluminal lesion. Ultrasound will reveal a rounded echogenic mass typically described as pseudokidney sign, while on CT scan an intussusception due to intestinal lipoma is seen as a well-circumscribed rounded homogenous lesion (Figure 2) with attenuation value between −40 and −120 HU, typical of fat [7].

For acute intussusception in adults, laparotomy is considered as a treatment of choice rather than making an attempt at hydrostatic reduction, as there is a considerable risk of an underlying malignancy [8]. Though the length of bowel involved with intussusception varies, controversy still prevails over the issue as to whether reduction of the intussusception should be tried intraoperatively. In the present case a preoperative diagnosis of an intraluminal lipoma as a cause for intussusception was made. As the lesion involved a considerable length of the small bowel an attempt to reduce the intussusception was made and was successfully accomplished. This saved a significant length of the normal small bowel from a major resection and thereafter its consequent morbidity. The segment containing the lipoma should then be resected. The only drawback of this method is that there may be inadvertent bowel injury and thereby a possibility of dissemination of malignant cells during manipulation while trying to reduce intussusception in cases of malignancy. Therefore one has to be gentle. If the attempt does not yield any result, it should be abandoned followed by a resection anastomosis [9].

## 4. Conclusion

Intussusception in adult is an uncommon condition invariably associated with an underlying cause which needs surgical intervention to arrive at a definitive diagnosis. Small bowel lipoma can cause intussusception presenting as obstruction. Ultrasound and CECT are very important adjuncts in preoperative diagnosis of intussusception. Early surgical intervention is the mainstay of definitive diagnosis and treatment.
